# Using big data and Population Health Management to assess care and costs for patients with severe mental disorders and move toward a value-based payment system

**DOI:** 10.1186/s12913-023-09655-6

**Published:** 2023-09-07

**Authors:** Valeria D. Tozzi, Helen Banks, Lucia Ferrara, Angelo Barbato, Giovanni Corrao, Barbara D’avanzo, Teresa Di Fiandra, Andrea Gaddini, Matteo Monzio Compagnoni, Michele Sanza, Alessio Saponaro, Salvatore Scondotto, Antonio Lora

**Affiliations:** 1 Center for Research on Health and Social Care Management, SDA Bocconi School of Management – Bocconi University, Via Sarfatti, 10, Milan, 20136 Italy; 2 Unit for Quality of Care and Rights Promotion in Mental Health, Istituto di Ricerche Farmacologiche Mario Negri IRCCS, Milano, Italy; 3 National Centre for Healthcare Research and Pharmacoepidemiology, University of Milano- Bicocca, Milan, Italy; 4 Unit of Biostatistics, Epidemiology and Public Health, Department of Statistics and Quantitative Methods, University of Milano-Bicocca, Milan, Italy; 5 General Directorate for Health Prevention, Ministry of Health, Rome, Italy; 6 Agency for Public Health, Lazio Region, Rome, Italy; 7 Department of Mental Health and Addiction Services, AUSL Romagna, Cesena, Italy; 8 General Directorate of Health and Social Policies, Emilia-Romagna Region, Bologna, Italy; 9 Department of Health Services and Epidemiological Observatory, Regional Health Authority, Sicily Region, Palermo, Italy; 10 Department of Mental Health and Addiction Services, ASST Lecco, Lecco, Italy

**Keywords:** Population health, Mental health, Healthcare delivery, Big data, Health information interoperability, Medical record linkage, Value-based healthcare, Mental health clustering tool

## Abstract

**Background:**

Mental health (MH) care often exhibits uneven quality and poor coordination of physical and MH needs, especially for patients with severe mental disorders. This study tests a Population Health Management (PHM) approach to identify patients with severe mental disorders using administrative health databases in Italy and evaluate, manage and monitor care pathways and costs. A second objective explores the feasibility of changing the payment system from fee-for-service to a value-based system (e.g., increased care integration, bundled payments) to introduce performance measures and guide improvement in outcomes.

**Methods:**

Since diagnosis alone may poorly predict condition severity and needs, we conducted a retrospective observational study on a 9,019-patient cohort assessed in 2018 (30.5% of 29,570 patients with SMDs from three Italian regions) using the Mental Health Clustering Tool (MHCT), developed in the United Kingdom, to stratify patients according to severity and needs, providing a basis for payment for episode of care. Patients were linked (blinded) with retrospective (2014–2017) physical and MH databases to map resource use, care pathways, and assess costs globally and by cluster. Two regions (3,525 patients) provided data for generalized linear model regression to explore determinants of cost variation among clusters and regions.

**Results:**

Substantial heterogeneity was observed in care organization, resource use and costs across and within 3 Italian regions and 20 clusters. Annual mean costs per patient across regions was €3,925, ranging from €3,101 to €6,501 in the three regions. Some 70% of total costs were for MH services and medications, 37% incurred in dedicated mental health facilities, 33% for MH services and medications noted in physical healthcare databases, and 30% for other conditions. Regression analysis showed comorbidities, resident psychiatric services, and consumption noted in physical health databases have considerable impact on total costs.

**Conclusions:**

The current MH care system in Italy lacks evidence of coordination of physical and mental health and matching services to patient needs, with high variation between regions. Using available assessment tools and administrative data, implementation of an episodic approach to funding MH could account for differences in disease phase and physical health for patients with SMDs and introduce performance measurement to improve outcomes and provide oversight.

**Supplementary Information:**

The online version contains supplementary material available at 10.1186/s12913-023-09655-6.

## Highlights

Mental health (MH) care often exhibits uneven quality and poor coordination of physical and mental health needs, especially for patients with severe mental disorders. Diagnosis alone may poorly predict condition severity and resources necessary to treat this patient group, but instruments such as the Mental Health Clustering Tool (MHCT) have been developed to better assess disease severity and diverse health needs within MH diagnosis groups.

A Population Health Management approach using administrative health databases, the Mental Health Clustering Tool, and diverse comorbidity measures was tested in three regions in Italy for patients with severe mental disorders to map delivery, consumption, and cost patterns, and explore determinants of variation in cost, aimed to better assess organization and quality of care.

Considerable variation in consumption patterns and costs, after stratifying patients based on needs and disease severity (MHCT) versus mere diagnosis, highlighted areas to address in designing a performance- versus volume-based payment model to better serve and follow MH patients. Methodology tested here using big - and “small” (from clinicians/patients) - data can be implemented by other healthcare systems to compare care pathways and outcomes across geographical areas and tie performance to payments for MH services.

**Supplementary Information:**

The online version contains supplementary material available at 10.1186/s12913-023-09655-6.

## Background

Mental health (MH) care is often characterized by uneven quality, high service use, problems with equitable access, and difficulties in coordinating care and addressing physical as well as mental health issues, especially for patients with severe mental disorders [1–4]. Population health management (PHM) is a patient-centred, data-driven approach to optimising the health of populations over individual life spans that begins with the identification of the population, assessment of their needs and risk stratification, and moves on to defining organizational and tailored interventions in a person-centered model [5, 6]. PHM interprets care as the ability to manage the continuum of health services through different phases of disease (i.e., prevention, diagnosis, treatment, follow-up, end-of-life care) to address the health needs of defined group of people at all points, stressing disease prevention, integration of social and health care, innovation in care delivery, and continual monitoring of the status of at-risk patients [5, 6]. By focusing on the health needs of a specified population, PHM can integrate and pursue both the Triple Aim (i.e., simultaneously improve population health and outcomes, and reduce costs) [7] and value-based care, that is, maximizing outcomes while minimizing costs (e.g., through integrating care, measuring outcomes and costs, moving to bundled payments) [8]. PHM often builds on efforts to integrate care to guide implementation and has been employed for various chronic conditions such as diabetes and congestive heart failure, notably in the United Kingdom (UK) [9, 10] and the United States [11–13]; however, its application in MH has been modest [4, 5, 7].

The aim of this study was to test the applicability of a PHM framework for MH using administrative health information systems to identify problems, evaluate care pathways and factors influencing costs in order to improve care, access and outcomes in a universal healthcare system (Italy) characterized by planning and funding allocation at the national level and decentralized care organization and delivery at the regional and local levels. We also tested use of the Mental Health Clustering Tool (MHCT), a needs assessment tool developed in the United Kingdom (UK) to better stratify patients according to severity and changing MH needs over time [14, 15]. Employing a holistic and multidisciplinary approach, independent of the diagnosis, the MHCT assigns patients that present similar characteristics to homogeneous severity groups, where patient needs of varying intensity and impact can be combined and analysed as episodes of care [14, 15]. Combined with physical health needs as evidenced in administrative health databases (AHDs), the study further aimed to provide a first step in exploring the feasibility of a bundled payment system to replace the current fee for service (FFS) reimbursement system in Italy to promote better outcomes and integration of care along the continuum for MH patients, especially those affected by severe mental disorders (SMDs).

Italy makes an interesting case study for its extensive system of health data collection (for physical and mental health) and its PHM approach to chronic disease management under the 2016 National Chronicity Plan [16]. A pioneer in the shift from institutionalism to community-based care through a major 1978 health reform, Italy has been a harbinger of radical change in MH care systems in Europe and the United States [17–19]. The resulting Italian organization of MH services into Mental Health Departments (MHDs) led to the development of intra-organizational (MH services) and extra-organizational (social and healthcare) collaboration, but also resulted in increased variation among MHDs and subsequently weakened integration of services and limited managerial models for performance evaluation/reporting [19, 20].

## Methods

### Data sources

Four MHDs from three Italian regions (Emilia-Romagna, Lazio and Sicily) participated in this pilot project, which was sponsored by the Italian Ministry of Health and the National Center for Disease Control and Monitoring (Centro Controllo Malattie) to explore geographic variations in care and the feasibility of introducing bundled payments to replace fees-for-service using available health data to guide the process. Data from the National Mental Health Information System (SISM, Sistema informativo salute mentale) tracks service use, socio-demographic and diagnostic characteristics of patients in Italy served by public, locally-based MHDs and private accredited providers funded through the National Health Service (NHS). According to the most recent Ministry of Health report on 2019 data, the nationwide SISM tracks more than 826,000 users (roughly 314,000 incident users), with a standardized prevalence rate of 164.5 per 10,000 adult inhabitants [21]. Data includes patient demographics (e.g., age, gender, years in the system), diagnoses (using the Ninth and Tenth Revisions, Clinical Modification, of the International Classification of Diseases, ICD9-CM and ICD10-CM), and MH services provided at the community, hospital, semi-residential and residential levels, and can be linked at the patient level to regional AHDs to track physical health service consumption using blinded, unique identifiers.

In SISM, MH residential facility use is recorded as length of stay in days, semi-residential as half-days, and community services are recorded by date, type of provider and service. AHDs included hospital discharge records with diagnoses (coded using ICD9-CM); publicly-financed medications (using Anatomical Therapeutic Chemical (ATC) codes); emergency department care, ambulatory care, and patient fee-exemption status. Additional databases, e.g., hospice, nursing homes, rehabilitation facilities, were not included in the analysis for lack of availability. Primary care is not tracked in AHDs in Italy, and was thus not included.

### Case identification and stratification

A random sample of patients with SMDs using MH services were selected (in order of presentation) from four MHDs in the three regions and assessed between January-March 2018 by healthcare professionals, upon completion of training, using version 5.0 of the MHCT Booklet to assign patients to one of 21 Clusters (Table 1) [15]. Application of the MHCT was carried out in four phases: preparation of the Italian version of the MHCT and related instructions for use; training of mental health professionals at participating facilities in the administration of the MHCT in accordance with methodology outlined in the MHCT Booklet; data collection and cluster allocation; and data analysis. Between October and December 2017, a total of 447 professionals were trained to use the MHCT in two-day sessions by two experts (AB and BD). The training involved 218 doctors, 152 nurses, 39 psychologists, 28 occupational therapists/educators, 7 social workers, and 3 other professionals. After the allocation to the super classes, each case was assigned to the best fitting cluster, using version 2.5 of the Technical Guidance for the MHCT assessment algorithm (https://assets.publishing.service.gov.uk/government/uploads/system/uploads/attachment_data/file/214910/Mental-Health-clustering-support-tool-algorithm.pdf).

The degree of probability of allocation of cases to the best fit cluster varied greatly across clusters. A probability of more than 50% was considered a correct allocation, while a probability of allocation lower than 50% identified the so-called “weak” clusters. The following diagnostic groups were considered SMDs: schizophrenia spectrum disorders (ICD9 codes 295, 297, 298; ICD10 codes F20-F29); major depression (ICD9 codes 296, 296.2, 296.3, 296.9; ICD10 codes F32-F39); bipolar disorders (ICD9 codes 296.0–1; 296.4-8; ICD10 codes F30-F31) and personality disorders (ICD9 -codes 301; ICD10 codes F60-F69).

**Table 1:** Definitions of the clusters for the Mental Health Clustering Tool

Relationship of care clusters to each other		Cluster	Definition	Complexity/ Need Ranking*		Recommended cluster review period
Non-psychotic	Mild/ Moderate/ Severe	1	Common mental health problems of low severity		19	12 weeks
		2	Common mental health problems of low severity but greater need		18	15 weeks
		3	Non-psychotic disorders of moderate severity		17	6 months
		4	Severe non-psychotic disorders		15	6 months
	Very severe and complex	5	Very severe non-psychotic disorders		12	6 months
		6	Non-psychotic disorders of over-valued ideas		14	6 months
		7	Enduring non-psychotic disorders (high disability)		11	Annually
		8	Non-psychotic chaotic and challenging disorders		9	Annually
Psychotic	1st episode	10	First episode of psychosis		6	Annually
	Ongoing or recurrent	11	Ongoing recurrent psychosis of low symptomatology		13	Annually
		12	Ongoing recurrent psychosis with high disability		8	Annually
		13	Ongoing recurrent psychosis of high symptomatology and high disability		3	Annually
	Psychotic crisis	14	Psychotic crisis		2	4 weeks
		15	Severe psychotic depression		4	4 weeks
	Very severe engagement	16	Psychosis and affective disorder (high substance misuse and engagement)		5	6 months
		17	Psychosis and affective disorder difficult to engage		1	6 months
Organic	Cognitive impairment	18	Cognitive impairment (low need)		20	Annually
		19	Cognitive impairment or dementia complicated (moderate need)		16	6 months
		20	Cognitive impairment or dementia complicated (high need)		10	6 months
		21	Cognitive impairment or dementia (high physical or engagement needs)		7	6 months

*Ranking from 1 (Highest complexity/need) to 20 (Lowest complexity/need). Cluster 9 is blank in the methodology. Adapted from the Mental Health Clustering Booklet, V5.0 (2016/2017) [9]

### Health assessment, consumption and costs

Using a blinded, unique identification number for each patient in the identified cohorts, a record-linkage procedure merged information provided by the MHCT with retrospective data retrieved from regional SISM and other AHDs for the years 2014–2017. Emilia-Romagna provided complete data for 2014–2017, but Lazio data was incomplete for 2017, and for both Sicily and Lazio, AHD but not SISM data was available for 2014. Therefore, comparative descriptive analysis across regions for consumption patterns was carried out only on 2015 and 2016 data, which was complete for all databases for the four MHDs, with 2016 used as an example of the latest year available. Consumption patterns and costs were calculated per cluster, while patient characteristics and comorbidities were tested for use in risk adjustment. Two methodologies were used to calculate comorbidities for the patient cohorts: the Charlson Comorbidity Index (CCI) and the multisource comorbidity score (MSC) [22, 23]. For CCI, diagnostic codes other than those in the SISM were only available for those patients who had either a hospitalization or an emergency room visit. Any diagnostic codes available in any years were used to calculate comorbidities.

MH and physical health service consumption was divided by type into specialized (e.g., psychiatric) consumption, including those services and procedures directly connected with MH (i.e., hospitalization for a psychiatric primary diagnosis in a psychiatric ward or ambulatory care coded as MH services), and non-specialized - or physical health - consumption, which included all admissions, medications, services and procedures connected with other disciplines. The care continuum was mapped through time, including both MH and physical health services. Costs (using reimbursement fees as a proxy) were calculated for delivering care per patient per year for each region.

Two regions (Lazio and Sicily) allowed data pooling for multivariate regression analysis using a generalized linear model (GLM), employed to estimate conditional mean costs and explore determinants of cost variation among and within clusters and regions. Emilia-Romagna, instead, denied access to the data for regression analyses for cost and computing time requirements (regional personnel were no longer available and centralized servers were over-burdened); and pooling with the other two regions’ data was not allowed. Complete retrospective data for both Lazio and Sicily was available only for the years 2015 and 2016 (see above). GLM assuming a gamma error distribution with log link was employed to account for the skewed distribution of healthcare costs with positive values, and few or no zero values. Panel regression was tested and results did not perceivably change. Analysis was performed using SAS (Version 9.0) for data management and descriptive statistics and Stata (version 17) for regression analysis.

## Results

Roughly 74,000 patients were identified from each of the three regional SISM databases, totalling 222,587. Patients over 18 years with SMDs numbered 29,570 (13.3%); a cohort of 9,019 patients (30.5%) underwent MHCT assessment and were included in the study database.

### Patient identification and stratification

**Fig. 1:**
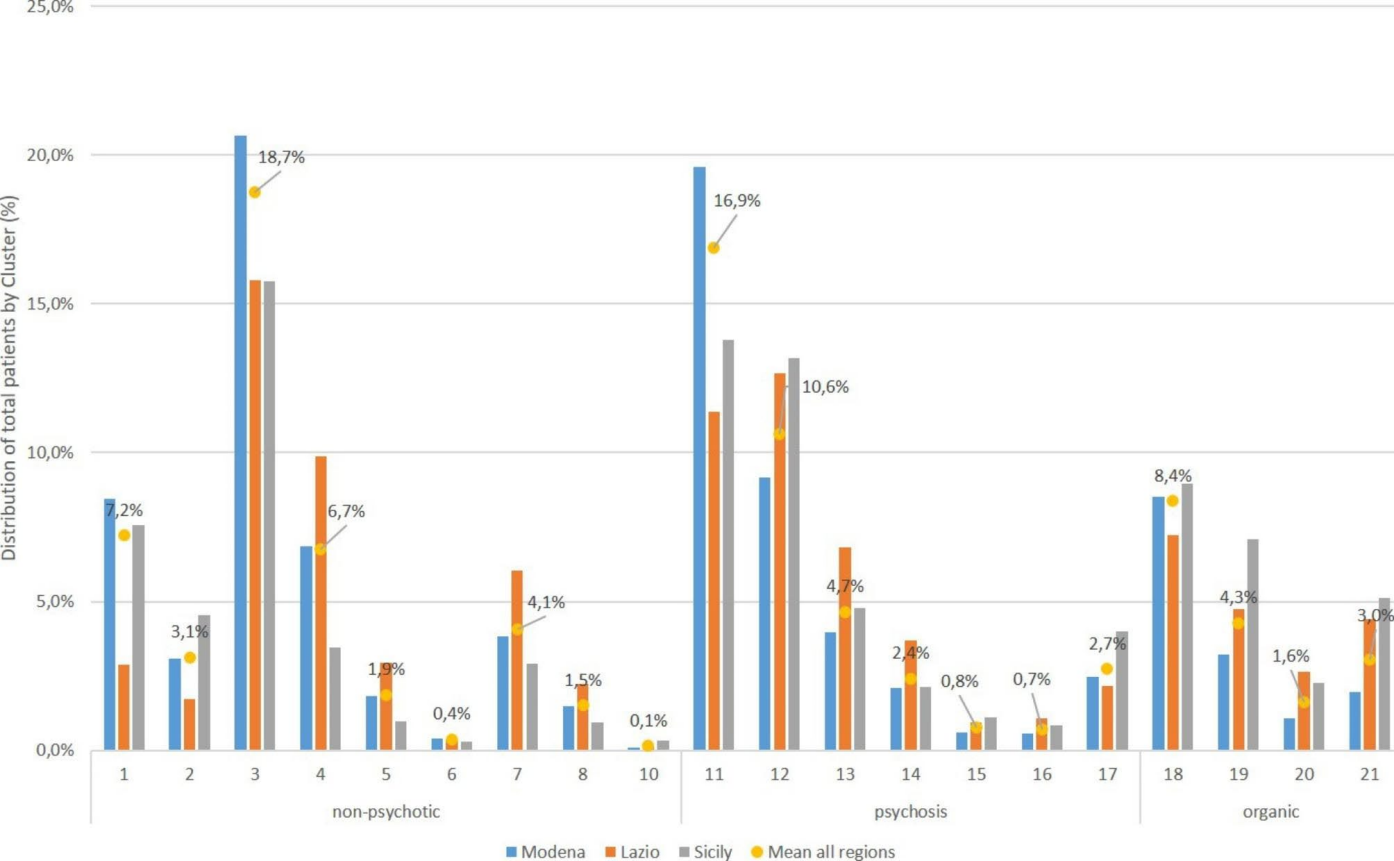
Patient distribution by Mental Health Clustering Tool cluster (numbered) and region (% of total patients), and combined average over all regions

Patient characteristics are illustrated in Table 2, while Fig. 1 illustrates distribution of patients by percentage among the MHCT clusters. Roughly 20% of database patients live in Sicily (MHD Palermo), 19% in Lazio (MHD Rome 2), and the largest group (61%) in Emilia-Romagna (MHDs Bologna and Modena). Characteristics of the three cohorts are similar in terms of number of years in the SISM (between 11.3 and 12.9 years) and the probability of correct assignment to one of the 21 clusters (0.73 to 0.76). Distribution of the population among age groups and mean age were fairly similar, with Emilia-Romagna patients slightly older, whereas the proportion of males differed considerably for Sicily (60.8% compared to 46.7% for Emilia-Romagna and 49.2% for Lazio). The two comorbidity measurement methodologies [22, 23] reveal a higher prevalence and severity of comorbidities for patients in Emilia-Romagna. All categories for both methodologies were measured, with a selection of the most frequently observed comorbidities presented in Table 2. Searching drug (ATC) codes as well as diagnostic codes (MCS methodology) captures greater numbers of patients with those conditions where CCI and MCS overlap (Table 2), e.g., the measured prevalence of diabetes was 5.8% in Emilia-Romagna using CCI, but rose to 12.8% using MCS.

**Table 2:** Patient characteristics for the cohort identified and assessed (Mental Health Clustering Tool), 2018

	Emilia-Romagna		Lazio		Sicily	
N. patients	5494		1703		1822	
Age groups	N.	%	N.	%	N.	%
age 18–24	208	3.8%	85	5.0%	56	3.1%
age 25–34	485	8.8%	193	11.3%	245	13.5%
age 35–44	934	17.0%	293	17.2%	449	24.6%
age 45–64	2860	52.1%	899	52.8%	921	50.5%
age 65–74	709	12.9%	184	10.8%	130	7.1%
age 75–84	268	4.9%	45	2.6%	19	1.0%
age over85	29	0.5%	4	0.2%	2	0.1%
	mean (sd)	min; max	mean (sd)	min; max	mean (sd)	min; max
Age	51.5 (14.1)	18; 97	49.2 (13.8)	18; 87	47,4 (12,4)	18; 86
Male	46,7%		50.6%		60,8%	
Time in the SISM*	11.3 (9.1)	0; 72	12.3 (8.7)	0; 70	12.9 (9.1)	0; 54
Probability correct MHCT* cluster allocation	0.76 (0.20)	0.76 (median)	0.74 (0.20)	0.78 (median)	0.73 (0.20)	0.72 (median)
Comorbidity measures	mean (sd)	min; max	mean (sd)	min; max	mean (sd)	min; max
Weighted charlsum*	0.36 (0.92)	0; 7	0.28 (0.82)	0; 6	0.29 (0.79)	0; 8
Charlson index (range 0–2)	0.29 (0.62)	0; 2	0.23 (0.54)	0; 2	0.24 (0.53)	0; 2
MCS* (overall score)	12.5 (8.4)	0; 68	7.4 (7.8)	0; 53	9.8 (7.0)	0; 51
MCS (class, from 1–5)	2.95 (1.3)	1; 5	2.1 (1.2)	1; 5	2.6 (1.2)	1; 5
MCS class 1, (%)	16%		41%		23%	
MCS class 2, (%)	24%		25%		25%	
MCS class 3, (%)	27%		21%		32%	
MCS class 4, (%)	18%		7%		12%	
MCS class 5, (%)	15%		6%		8%	
Comorbidities most frequently observed among patients (%, by methodology)						
	Charlson	MCS	Charlson	MCS	Charlson	MCS
Diabetes	5.8%	12.8%	3.3%	8.5%	7.5%	14.2%
COPD*	4.6%		4.7%		3.4%	
Cerebrovascular disease	4.1%		3.1%		3.6%	
Congestive heart failure	2.2%	12.9%	1.0%	7.9%	1.1%	12.5%
Hypertension		36.3%		20.3%		30.8%
Pulmonary disease		31.1%		16.4%		30.6%
Ulcers		11.4%		11.3%		23.3%

*SISM - Sistema informativo salute mentale (National Mental Health Information System); MHCT – Mental Health Clustering Tool; Charlsum is the weighted score from all 17 categories of comorbidities included in the Charlson Comorbidity Index methodology [16], which assigns a score of 1, 2 or 6 to each category; MCS – Multisource comorbidity score [17]; COPD – chronic obstructive pulmonary disease

Patient distribution is highly heterogeneous both among clusters within the same region and within the same cluster across regions (Fig. 1). Overall, Cluster 3 (Non-psychotic - moderate severity, 18.7%), and Ongoing recurrent psychosis of low symptomatology (Cluster 11, 16.9%) or high disability (Cluster 12, 10.6%) were most frequently observed. Nearly half of patients in each superclass belonged to a single cluster: Cluster 3 (43%) for non-psychotic, Cluster 11 (43.4%) for psychotic and Cluster 18 (cognitive impairment, low need, 48.4%) for organic. All three of these clusters represent low to moderate needs.

### Health assessment and resource consumption patterns

**Fig. 2:**
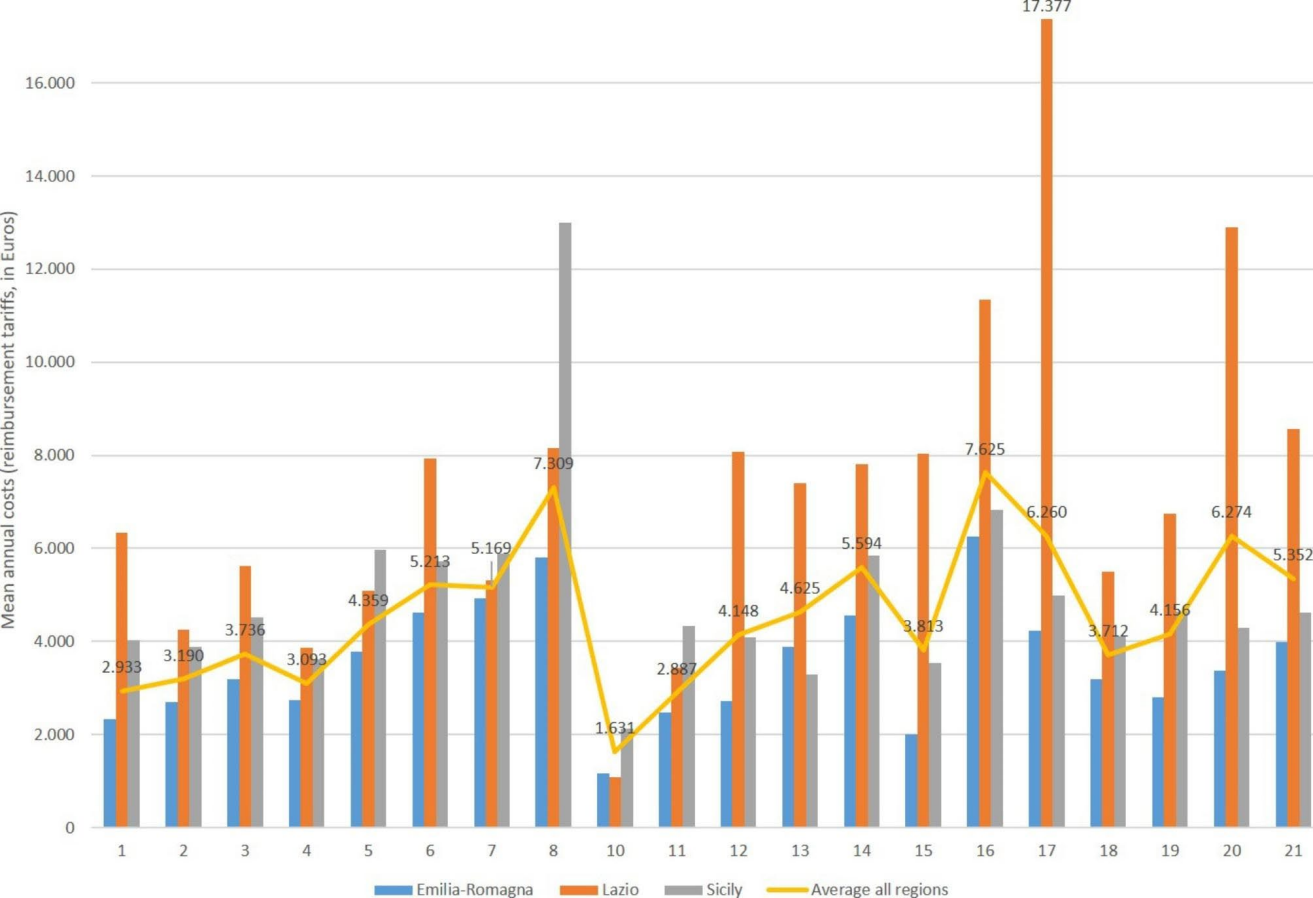
Mean costs (reimbursement tariffs, in Euros) per patient by Mental Health Clustering Tool cluster (numbered) and region, Year 2016

The analysis of consumption patterns for each region and cluster allows for identification of the active caseload for any financial year, defined as the number of services used by patients who received treatment or assessment. The active caseload and the annual mean costs per patient (using reimbursement fees as a proxy) of delivering care per region and cluster for the sample year 2016 are shown in Table 3a and Table 3b and Fig. 2, where variations among clusters and regions (notably between Clusters 8, 16, 17, 20) are observed. Annual mean per patient costs for the 8,469 patients observed in the databases in 2016 were €3,925 (€1,445, or 37%, for services noted in the SISM database and €2,480, 63%, in the physical health databases). Emilia-Romagna had the lowest mean costs of the three regions (€3,101), Lazio was highest at €6,501, and Sicily registered mean costs of €4,434.

**Table 3:** a. Mental health and non-mental health service consumption, 2016, per region, all clusters. Number, percentage of patients and mean costs (reimbursement) per patient by consumption in the various databases for mental health (SISM databases) and physical health (AHD) databases

	Numbers of patients by database			% of patients by database		
Region*	E-R	L	S	E-R	L	S
**N. Patients**	5,350	1,362	1,757	63%	16%	21%
SISM* Residential facility patients (pts), N.,%	311	161	48	6%	12%	3%
SISM Semi-residential facility patients, N., %	157	103	97	3%	8%	6%
SISM ambulatorial services pts, N., %	4,653	1,326	1,543	87%	97%	88%
AHD* Hospital patients, N., %	1,171	256	408	22%	19%	23%
AHD Emergency department (ED) pts, N., %	1,979	448	439	37%	33%	25%
AHD ambulatory services pts. N., %	4,553	1,062	1,215	85%	78%	69%
AHD Medication purchases patients, N., %	5157	1099	1526	96%	81%	87%
Annual mean per patient costs (reimbursement), €	3,101	6,501	4,434			
SISM database mean per patient costs, % of total mean costs	510	3,971	2,332	16%	61%	53%
AHD costs, mean, % of total mean costs	2,591	2,530	2,102	84%	39%	47%

*Regions, E-R Emilia-Romagna, L-Lazio, S-Sicily; SISM - Sistema informativo salute mentale (National Mental Health Information System), AHD-Administrative Health Databases

**Table 3:** b. Consumption by mean volume, split between services for mental health and services for physical health with the overall percentage of costs for each category, 2016, per region, all clusters

Region*	E-R	L	S
N. Patients, total and per cluster	5,350	1,362	1,757
Specialized consumption (mental health - psychiatric), mean volume per patient			
SISM N. days, residential facility	2.4	17.2	6.5
SISM N. days, semi-residential facility	0.9	1	0.9
SISM N. days with ambulatory services use	17.3	5.8	4.6
AHD N. hospitalizations in psych wards	0.2	0.2	0.4
AHD N. outpatient psych services	0.1	0.7	0.1
AHD N. prescriptions for psychiatric medications (ATC N class)	26.3	25.5	27.8
AHD emergency visits for psychiatric services	0	0	0.1
% of total costs for specialized services	57%	86%	81%
Non-specialized (physical health, non-psichiatric) consumption, mean volume per patient			
AHD N. hospitaliz. non-psychiatric wards	0.2	0.1	0.1
AHD N. all other outpatient services	19.1	18.9	16.4
AHD, N. prescriptions other ATC classes	12.6	18.5	21.1
AHD non-psych ED visits	0.9	0.8	0.7
AHD N. all other outpatient services	19.1	18.9	16.4
AHD, N. prescriptions other ATC classes	12.6	18.5	21.1
AHD non-psych ED visits	0.9	0.8	0.7

*Regions, E-R Emilia-Romagna, L-Lazio, S-Sicily; SISM - Sistema informativo salute mentale (National Mental Health Information System), AHD-Administrative Health Databases

Using 2016 as an example, Table 3a shows the number, percentage of patients and mean costs per patient by consumption for all cases in each region while Table 3b shows the consumption by mean volume, split between services for mental health and services for physical health. Table 3c shows the split for three sample clusters with the highest numbers of patients in each superclass (Clusters 3, 11, and 18). Consumption of specialized versus non-specialized services seems to show more variation across regions. While difficult to quantify the split in the number or volume of total services, the costs (reimbursement tariffs) for specialized services (for psychiatric/psychological care in AHDs and all SISM services) accounted for 57% of total costs for Emilia-Romagna, 86% for Lazio and 81% for Sicily. Variation among regions and clusters is notable, particularly in the use of residential and semi-residential facilities, e.g., in Lazio some 12% of the 1362 patients identifiable in the various databases for that year spent at least some time in residential MH facilities, compared to only 6% for Emilia-Romagna and 3% for Sicily. The proportion of hospitalizations (AHD) in psychiatric wards, however, was higher for Sicily.

**Table 3:** c. Consumption by mean volume, split between services for mental health and services for physical health for three sample clusters, per region, 2016

	Cluster 3				Cluster 11					Cluster 18				
Region*	E-R	L	S			E-R	L	S			E-R	L	S	
N. Patients, total and per cluster	1,119	208	277			1,046	152	247			463	104	157	
Specialized consumption (mental health - psychiatric), mean volume per patient														
SISM N. days, residential facility	2	10.4	4.8			1.3	5	5.6			1.8	13.4	1	
SISM N. days, semi-residential facility	0.8	3.6	2.9			0.9	0.7	6.8			0.9	6.6	10.3	
SISM N. days with ambulatory services use	15.4	3.9	3.2			15	0.7	7			17.2	6.8	10.6	
AHD N. hospitalizations in psych wards	0.2	0.2	0.3			0.1	0.2	0.3			0.2	0.1	0.3	
AHD N. outpatient psych services	0.1	0.6	0.1			0.1	0.8	0.1			0.1	0.6	0.1	
AHD N. prescriptions for psychiatric medications (ATC N class)	25.9	22.7	24.5			22.5	20.1	26.4			25.6	24.4	28.3	
AHD emergency visits for psychiatric services	0	0	0.1			0	0	0			0	0	0	
% of total costs for specialized services	52%	82%	69%			49%	86%	85%			55%	87%	89%	
Non-specialized (physical health, non-psichiatric) consumption, mean volume per patient														
AHD N. hospitaliz. non-psychiatric wards	0.2	0.1	0.1			0.2	0	0.1			0.1	0.1	0.1	
AHD N. all other outpatient services	21	23.4	16.8			17.5	15.1	15.3			20.3	21.1	16.3	
AHD, N. prescriptions other ATC classes	13.2	22.6	24.5			11.6	13	18.7			15.6	20.8	18.7	
AHD non-psych ED visits	0.9	0.6	0.5			0.7	0.6	0.6			0.8	0.7	0.8	
AHD N. all other outpatient services	21	23.4	16.8			17.5	15.1	15.3			20.3	21.1	16.3	
AHD, N. prescriptions other ATC classes	13.2	22.6	24.5			11.6	13	18.7			15.6	20.8	18.7	
AHD non-psych ED visits	0.9	0.6	0.5			0.7	0.6	0.6			0.8	0.7	0.8	
Annual mean per patient costs (reimbursement), €	3,193	5,628	4,512			2,464	3,436	4,344			3,180	5,497	4,100	
SISM database mean per patient costs	456	2,852	1,994			360	1,510	2,627			420	3,534	2,558	
SISM database costs, % of total mean costs	14%	51%	44%			15%	44%	60%			13%	64%	62%	
AHD costs, mean	2,736	2,776	2,518			2,104	1,926	1,717			2,760	1,963	1,543	
AHD costs, % of total mean costs	86%	49%	56%			85%	56%	40%			87%	36%	38%	

*Regions, E-R Emilia-Romagna, L-Lazio, S-Sicily; SISM - Sistema informativo salute mentale (National Mental Health Information System), AHD-Administrative Health Databases

Variation is also notable among clusters, where even those characterized by moderate need or symptomatology (Clusters 3 and 11) showed higher use of residential facilities and higher shares of costs for specialized services in Lazio and Sicily compared to Emilia-Romagna. As expected, ambulatory care use of specialized services was minimal, in keeping with high percentages of patients using SISM ambulatory services, though use in Sicily was considerably lower, 69% compared to 85% and 78% for the other two regions. Among those not shown, clusters 13–17 (characterized by severe, crisis or high disability needs) averaged across the three regions 83% for the portion of costs for specialized services, considering all databases.

Factors that affect costs can be discerned from regression results (Table 4), using the pooled 2015–2016 data from Lazio and Sicily. In Model 1, controlling for sex, year, region, age, time in SISM database, probability of correct MHCT assignment, and MCS comorbidity classes, residency in Lazio (versus Sicily) raised costs by more than 40%, and age classes, compared to the 45 to 64 years age class, showed significance for all groups: younger patients cost between 25 and 48% more than 45–64 year-olds, whereas groups over 65 cost 21–32% less. Considering comorbidity, compared to patients with few or no comorbidities (MCS class 1), rising by one class raises costs by 73%, while two classes above (MCS 3) more than doubles costs, MCS 4 increases costs nearly three-fold and MCS 5 raises costs nearly 4-fold compared to MCS 1. Time in SISM was significant but had virtually no effect on costs for each additional year. Across models, the specific diagnosis (by ICD9/10 code) had no significant effect.

**Table 4:** Factors that determine costs, patients with severe mental disorders, pooled data (Sicily, Lazio), 2015–2016

Model*	1	2	3	4	5
Independent variables					
Male	1.075	1.04	0.985	1.004	1.000
Lazio	1.443^***^	1.502^***^	1.403^***^	1.056	1.066^*^
Year 2015	1	1	1	1	1
Year 2016	1.004	0.997	0.991	0.957	0.955
18–24 years	1.364^*^	1.305^*^	1.365^*^	1.124	1.135
25–34 years	1.478^***^	1.423^***^	1.353^***^	1.074	1.071
35–44 years	1.253^***^	1.224^***^	1.238^***^	1.043	1.044
65–74 years	0.790^**^	0.825^*^	0.825^*^	1.04	1.041
over 74 years	0.683^*^	0.711^*^	0.667^**^	1.041	1.037
MCS class 1	1	1		1	1
MCS class 2	1.728^***^	1.684^***^		1.079	1.068
MCS class 3	2.068^***^	2.046^***^		1.146^**^	1.136^**^
MCS class 4	2.881^***^	2.932^***^		1.410^***^	1.400^***^
MCS class 5	3.756^***^	3.829^***^		1.647^***^	1.644^***^
Time in MH database (SISM)	1.010^***^	1.007^**^	1.009^***^	1.008^***^	1.008^***^
Probability of correct MHCT	0.959	0.994	0.966	0.982	0.987
Hospitalization				2.514^***^	2.516^***^
Emergency room visit				0.968	0.973
Ambulatory services				1.397^***^	1.404^***^
Medication purchases				1.638^***^	1.639^***^
Territorial MH services				2.031^***^	2.019^***^
Semi-residential MH care				4.514^***^	4.510^***^
Residential MH care				8.164^***^	8.144^***^
Schizophrenia		1.117	1.073		1.093
Major depression		0.831	0.857		1.017
Bipolar disorder		0.882	0.926		1.126
Personality disorder		1	1		1
Constant	1792.5***	1794.7***	3700.9***	464.5***	434.8***
*Observations*	6180	6180	6180	6180	6180

Exponentiated coefficients; *p*-values: ^*^p < 0.05, ^**^p < 0.01, ^***^p < 0.001

Abbreviations: MCS ? Multisource comorbidity score; MHCT ? Mental Health Clustering Tool; SISM- Sistema informativo salute mentale (National Mental Health Information System)

Models (1- Basic – controlling for gender, region, year, age class, comorbidities, 2-Basic plus diagnoses, 3-Basic excluding comorbidities; 4 and 5-Basic plus presence in various data flows, without (4) and with (5) diagnoses

Adding controls for service consumption in the various databases (dummy variables equal to 1 if present in the database, 0 otherwise) erased significance for age effects; comorbidity classes retained significance though the effects were considerably lessened. The presence of at least one service consumed in the physical health administrative databases (with the exception of emergency care), significantly raised costs, with the largest effect observed for hospital admissions. MH care use showed residential and semi-residential care consumption to raise costs by more than 4 and 8 times, respectively. Supplementary Figure S1 reports crude mean costs compared to adjusted (using non-linear prediction) mean costs (95% confidence intervals) from regression analysis for three of the models (1, 2 and 5), showing considerable effects when adjusting for administrative database flows. Supplementary Table S1 reports regression results on SISM costs, AHD costs and selected clusters. Panel regression was performed to test the model (not shown), and results were virtually unaltered.

## Discussion

This study has explored the use of big data within a PHM approach, using mental health and administrative healthcare databases from four mental health departments in three regions in Italy, to assess its potential for planning and evaluating MH care services, costs, pathways and interventions, with an underlying goal to explore a move toward a value-based, prospective payment system.

As largely discussed in the literature,[24, 25] AHDs from national or regional health systems constitute a fundamental source of readily available and relatively inexpensive, large quantities of data, reliable for their population coverage and collection over time, especially in countries with a NHS (e.g., Italy, the United Kingdom, Canada, Finland) or with extensive systems of electronic health records and claims data (such as the United States and Germany). Such databases typically contain information regarding healthcare organizations (e.g., hospitals, ambulatory care, emergency care, long-term care) and patients (including demographics and clinical diagnosis and procedure codes) as these pertain to specific clinical encounters that might be useful in monitoring quality of care, targeting disparities in access, and understanding consumption and cost patterns throughout the care pathway (community vs. hospital settings, professionals involved, key phases in the episode of care, service use). The routine collection of data and evaluation of the quality of the care provided in its various facets (appropriateness, continuity, safety and effectiveness) is important for patients with SMDs, particularly since they are associated with greater clinical and psychosocial needs and make up about two-thirds of adult MH service users in Italy [21]. Where evaluations of existing delivery models reveal problems for all or specific targets of patients, new delivery models can be proposed in an informed fashion and analysed to address questions of equity in access and outcomes. The use of big data and PHM approaches can additionally offer significant opportunities to develop new payment models for MH care, including bundled payments to allow for a shift from volume-based to value-based payment models [26].

As highlighted by a realist literature review which underlies this study, [27] developing episodic or bundled payments to replace fee-for-service systems requires providers and commissioners to cover at least three elements. First, develop an understanding of the active caseload being served by the providers involved, defining for any financial year the number of services provided to the patients in each cluster, the case mix of services provided, and the relative costs. Second, determine the episode of care for each cluster and the delivery model for the specific target population in different contexts to explain possible underlying reasons for cost variability. Third, risk adjustment to adapt the bundled payment to patient characteristics (e.g. age, sex, comorbidities) and regional or local variation in healthcare services organization and financing. The analysis presented in this paper aimed to test this model, by identifying the population, analysing the consumption patterns and costs per cluster and the factors affecting cost variations among regions and among clusters within the same region. Within a value-based perspective, a further step would be to determine how quality and outcome measures (including also patient preferences and experience) should be linked to payment. These aspects should be further explored and monitored using appropriate metrics [28].

Quality of care for MH is often characterized by large geographic variability, both because of significant underinvestment in terms of attention and resources and because of the lack of standardization among organizations, mandates and care pathways [3, 29–31]. Analysis of the data here revealed a diversity of consumption models and costs for patients with severe mental disorders across MHDs and regions, which provide information on care provision and coordination that would be difficult to discern from clinical data or administrative data alone. Adding in the MHCT, based on clinical assessments, to stratify patients in the database helps to address the potentially poor predictive value of the diagnosis in defining the severity of the condition of MH patients and the resources necessary for care [32]. The MHCT was borrowed from the UK experiment in performance-based financing and is well-known in the UK among the community of psychiatrists and MH professionals [14, 33]. The segmentation of patients into 21 clusters according to the presenting problem severity and needs for care could provide a reliable proxy for patient needs and resource use instead of diagnoses since different levels of clinical and psychosocial involvement can be associated with the same diagnosis [33]. As such, the additional information based on the stage and severity of the condition provides insight into how critical differences in the mix and coordination of care could be addressed without compromising the level of severity of the disease (as performance measures).

For example, Emilia-Romagna appears to provide more care through ambulatory MH services as opposed to residential care, with lesser associated costs. As national policies emphasize territorial over residential care for MH patients [17–20, 29, 30], care organization in Sicily and Lazio (and other regions) might be improved in this area, and regular administration of the MHCT would allow for comparisons of which model better serves patients in better managing severity and improving outcomes. The variation in care pathways and costs also highlights problems across MHDs in coordinating care, which has been shown to worsen outcomes, including mortality, as observed in a French study [34]. While it is difficult to determine which combinations of care lead to the best outcomes, tools such as the continuity of care index used in that study and the MHCT used here could be incorporated into analysis of MH care in Italy. We recommend that future research make further use of the types of services consumed in the SISM territorial services database, linked to outcomes where possible, to help identify best practices and determine where care in residential facilities might best be replaced by territorial services.

Additionally, problems noted in the UK regarding assigning clusters accurately, defining episodes, appropriateness for different settings of care, and physician discretion in coding within a prospective payment system [28, 33, 35], along with the costs of training personnel and ongoing assessment in implementing the MHCT (or a similar tool) in Italy and elsewhere, would need to be considered. The methodology used here included translating the MHCT for use in Italy and comprehensive training of healthcare professionals, which makes it suitable for expansion across Italy; however, difficulties noted in correctly assigning clusters would need to be addressed and accounted for.

Mental health has been shown to significantly and negatively impact physical health (and vice versa), with implications for access to care [1, 2]. Our results go beyond the MHCT, showing the impact of the mix of services for physical and mental health on overall resource use and costs, providing evidence that health systems should use data to incorporate both aspects to better allocate resources and coordinate care to address issues surrounding poorer physical health outcomes for MH patients [1]. Moreover, though drivers of diversity in service utilization and outcomes across regions and countries have been studied in other diseases [36], few have explored these areas in MH, a gap our study addresses. The impact on costs registered here for comorbidities shows the importance of addressing and following chronic conditions in MH patients. As such, the techniques applied here coincide with a pillar of the PHM model within an integrated care model framework: the definition and implementation of tailored patient-centred interventions targeting specific patient groups [2, 5, 6, 37].

Finally, the Italian case is significant for its pioneering shift from institutional to community-based care, extensive availability and use of data, and population health framework, which has made it possible to track developments and difficulties over time [16, 19, 20]. Problems related to quality of care, access and uneven application of the reforms over time have led to projects such as that described here to expand the use of linkable information to provide a more comprehensive analysis of care and delivery, especially for patients with SMDs, and explore the feasibility of structured means to drive improvement, such as results-based funding (e.g., bundled payments) in the future.

### Limitations

Prerequisites for the adoption of a PHM approach is the availability of good quality data and the interchangeability of data [5, 24, 25]. The SISM in Italy has only recently achieved good quality, but some information may still be incomplete, and it lacks detailed information on treatment content and outcomes. We also had considerable difficulty in obtaining the same type, timeframe and quality of data for each region, and, while we noted considerable regional variation, other than the inclusion of regional fixed effects into the regression, we did not further account for socio-economic and cultural differences in the two regions (Lazio and Sicily), an important qualifier for analyses of the Italian NHS. Efforts should be made in future research to account for these factors, e.g., inclusion of macroeconomic measures such as regional income levels, population demographics, unemployment rates, which vary greatly among regions in Italy. Increased collaboration among MHDs in different regions on organization and coordination of care, linked to outcome measures, should also be encouraged in national and regional policy to better identify and share best practices.

Because the SISM and AHDs are based on the diagnostic code, and considering its poor predictive value for resource intensity and use, we conducted this pilot project to enable the use of the MHCT, which required individual administration of the tool and subsequent linkage to AHDs, not likely to be possible in all countries and settings. Additionally, the SISM does not cover office-based private practice, therefore limiting, albeit to a small extent, the sample representativeness. Private spending accounts for some 23% of total expenditures for healthcare in Italy (roughly 37.5 billion Euros in 2018 compared to 119.1 billion for public healthcare spending, 156.5 billion overall), and all types of ambulatory care, including co-payments for public services, accounted for about 14% of that (5.4 billion Euros) [38]. We did not have data on primary care (not collected in AHDs), however, General Practitioners do not provide MH services directly, but function largely as gatekeepers to specialist care. Additionally, no information is collected on the medical reasons for any visits.

Our study was also limited by its retrospective quality, which forced us to assume that cluster definition was applicable to past service use, and, though tested, we could make scarce use of clusters in regression analysis. Future research should explore the diversity of consumption models for the same cluster with follow-up data, ideally examining whether needs based on cluster assignment have been considered and met, looking at the differing interpretations of patient management adopted by regions and clinicians, particularly as regards the concept of deinstitutionalization of psychiatric patients and the available healthcare service supply. Though AHDs are being increasingly linked to clinical data, such techniques also raise important privacy questions [24, 25].

Despite great interest in the development and potential connected with PHM approaches, a mature understanding of PHM logic is hindered by the paucity of information and data on social dimensions [39]. Analyses are still concentrated mostly on the health dimension alone, but especially for MH, there is an important effect connected with social, economic and environmental dimensions [2]. In fact, linkage to social services databases was not possible here. A limitation of our study was the lack of data on social aspects, in particular in regression analysis to determine the drivers of variation. In addition, a move toward systematically measuring and incorporating patient reported outcomes in MH care, using a number of available scales and measures, is occurring in other countries, notably France [40], which we lacked here. We therefore recommend that future research and national and regional MH programs explore incorporating patient-reported outcome measures – and patient preferences - and linkage to social services databases in the future to incorporate social and other dimensions into the analysis.

## Conclusions

This study using mental and physical health administrative information systems uncovered considerable differences in consumption patterns and drivers of costs for patients with SMDs stratified using the MHCT in Italy, raising questions regarding the role of planning, providing and monitoring MH services across regions and the level of integration and coordination between services within and among regions. Analysis using big data and the PHM approach here showed the importance of considering both physical and MH, where regression analysis revealed the considerable impact on costs exerted by comorbidities, residential psychiatric care and physical healthcare consumption, providing new perspectives regarding system sustainability. Any move toward new payment schemes, e.g., bundled payments, should use the techniques tested here to focus on the most appropriate care delivered within a care episode or care program, either population or individually defined, at the lowest cost. PHM can promote innovative improvement interventions by building policies, delivery models, and system governance on the basis of precise data and information on consumption patterns and population, supported through continuous experimentation and maintenance.

The PHM approach tested here can be implemented by other healthcare systems at the national, regional or local level. How, and how completely, the components are implemented will depend largely on the specific characteristics of healthcare practices or organizations; the resources available to support the effort; the breadth and depth of existing information systems and privacy concerns; the level of skill and acceptance of information system management among policy makers, administrators and clinicians; and the collaborations and partnerships that exist within the matrix of the healthcare system.

### Electronic supplementary material

Below is the link to the electronic supplementary material.

**Supplementary Table S1**: 2015-2016 SICILIA AND LAZIO ? GLM regressions for all data and selected clusters for costs, SISM costs, AHD costs. Models (M1) Full model on costs, (M2) Full model on SISM costs, (M3) SISM costs no SISM flows (M4) Costs physical health datasets (AHD) (M5) Costs AHD no AHD flows (M6) Costs Cluster 3 (M7) Costs Cluster 4 (M8) Costs Cluster 11 (M9) Costs Cluster 12 (M10) Costs Cluster 13 (M11) Costs Cluster 18 (M12) Costs Cluster 19. **Supplementary Figure S1** _ Mean crude and adjusted (for models 1, 2 and 5) costs (reimbursement tariffs) and 95% confidence intervals, pooled Sicily and Lazio, years 2015-2016 - All and Clusters 3, 11, 12, 18, 19.

## Data Availability

The datasets presented in this article are not readily available because the data that support the findings of this study are available from the Regions of Lazio and Emilia-Romagna, and the Province of Palermo, but restrictions apply to the availability of these data, which were used under license for the current study, and so are not publicly available. Requests to access the datasets should be directed to the corresponding author.

## List of abbreviations

MH: Mental health
PHM: Population Health Management
MHCT: Mental Health Clustering Tool
SMD: Severe mental disorders
AHD: Administrative health databases
FFS: Fee For Service
MHD: Mental Health Departments
NHS: National Health Service
SISM: Mental Health Information System
ICD: International Classification of Diseases
ATC: Anatomical Therapeutic Chemical
CCI: Charlson Comorbidity Index
MSC: Multisource Comorbidity Score
GLM: Generalized linear model

## References

[1] Ronaldson A, Elton L, Jayakumar S, Jieman A, Halvorsrud K, Bhui K (2020). Severe mental illness and health service utilisation for nonpsychiatric medical disorders: a systematic review and meta-analysis. PLOS Med.

[2] Lawrence D, Kisely S (2010). Inequalities in healthcare provision for people with severe mental illness. J Psychopharmacol Oxf Engl.

[3] Institute of Medicine (US) Committee on Crossing the Quality Chasm (2006). Adaptation to Mental Health and Addictive Disorders. Improving the quality of Health Care for Mental and Substance-Use Conditions: Quality Chasm Series.

[4] Sieck CJ, Wickizer T, Geist L (2014). Population health management in integrated physical and mental health care. Adv Health Care Manag.

[5] Struijs JN, Drewes HW, Heijink R, Baan CA (2015). How to evaluate population management? Transforming the Care Continuum Alliance population health guide toward a broadly applicable analytical framework. Health Policy.

[6] Kindig D, Stoddart G (2003). What is Population Health?. Am J Public Health.

[7] Berwick DM, Nolan TW, Whittington J (2008). The Triple Aim: Care, Health, and cost. Health Aff (Millwood).

[8] Porter ME, Lee TH (2013). The strategy that will fix health care. Harv Bus Rev.

[9] Deloitte Centre for Health Solutions. The transition to integrated care. Population Health Management in England; 2019.

[10] NHS England » Population Health and the Population Health Management Programme. https://www.england.nhs.uk/integratedcare/what-is-integrated-care/phm/. Accessed 27 Apr 2023.

[11] Levitz C, Jones M, Nudelman J, Cox M, Camacho D, Wielunski A (2022). Reducing Cardiovascular risk for patients with diabetes: an Evidence-Based, Population Health Management Program. J Healthc Qual Off Publ Natl Assoc Healthc Qual.

[12] Schmittdiel JA, Gopalan A, Lin MW, Banerjee S, Chau CV, Adams AS (2017). Population Health Management for diabetes: Health Care System-Level Approaches for improving quality and addressing disparities. Curr Diab Rep.

[13] Khazanie P, Allen LA (2020). Systematizing heart failure Population Health. Heart Fail Clin.

[14] Jacobs R (2014). Payment by results for mental health services: economic considerations of case-mix funding. Adv Psychiatr Treat.

[15] Mental Health Clustering Booklet, V5.0. (2016/2017). NHS England Publications Gateway Reference 04421. https://assets.publishing.service.gov.uk/government/uploads/system/uploads/attachment_data/file/499475/Annex_B4_Mental_health_clustering_booklet.pdf. Accessed 11 Jan 2021.

[16] Ministero della Salute (Ministry of Health). Piano nazionale della cronicità (National Chronicity Plan), 2016. https://www.salute.gov.it/portale/documentazione/p6_2_2_1.jsp?id=2584&lingua=italiano.

[17] Dumont MP, Dumont DM (2008). Deinstitutionalization in the United States and Italy: a historical survey. Int J Ment Health.

[18] Mosher LR (1982). Italy’s revolutionary mental health law: an assessment. Am J Psychiatry.

[19] Fioritti A (2018). Is freedom (still) therapy? The 40th anniversary of the italian mental health care reform. Epidemiol Psychiatr Sci.

[20] Tozzi V, Pacileo G (2017). Salute mentale in Italia: Sfide e prospettive manageriali nella sanità che cambia.

[21] Ministero della Salute. Rapporto salute mentale: analisi dei dati del Sistema informativo per la salute mentale (SISM) anno 2019. 2021.

[22] Charlson ME, Pompei P, Ales KL, MacKenzie CR (1987). A new method of classifying prognostic comorbidity in longitudinal studies: development and validation. J Chronic Dis.

[23] Corrao G, Rea F, Di Martino M, De Palma R, Scondotto S, Fusco D (2017). Developing and validating a novel multisource comorbidity score from administrative data: a large population-based cohort study from Italy. BMJ Open.

[24] Salas-Vega S, Haimann A, Mossialos E (2015). Big Data and Health Care: Challenges and Opportunities for Coordinated Policy Development in the EU. Health Syst Reform.

[25] Raghupathi W, Raghupathi V. Big data analytics in healthcare: promise and potential. Health Inf Sci Syst. 2014;2. 10.1186/2047-2501-2-3 PMC4341817 25825667

[26] Bundled-Payment Models Around World. : How They Work, Their Impact | Commonwealth Fund. https://www.commonwealthfund.org/publications/2020/apr/bundled-payment-models-around-world-how-they-work-their-impact. Accessed 25 Mar 2021.

[27] Ferrara L, Tozzi VD. Bundled payment for mental health care: a realist review. Int J Integr Care; 2018. p. 155.

[28] Jacobs R, Chalkley M, Aragón MJ, Böhnke JR, Clark M, Moran V (2018). Funding approaches for mental health services: is there still a role for clustering? BJPsych. Adv.

[29] Lora A, Barbato A, Cerati G, Erlicher A, Percudani M (2012). The mental health system in Lombardy, Italy: access to services and patterns of care. Soc Psychiatry Psychiatr Epidemiol.

[30] Lora A, Conti V, Leoni O, Rivolta AL (2011). Adequacy of treatment for patients with schizophrenia spectrum disorders and affective disorders in Lombardy, Italy. Psychiatr Serv Wash DC.

[31] Asthana S, Gibson A, Bailey T, Moon G, Hewson P, Dibben C (2016). Equity of utilisation of cardiovascular care and mental health services in England: a cohort-based cross-sectional study using small-area estimation.

[32] Maj M (2020). Beyond diagnosis in psychiatric practice. Ann Gen Psychiatry.

[33] Trevithick L, Painter J, Keown P (2015). Mental health clustering and diagnosis in psychiatric in-patients. BJPsych Bull.

[34] Hoertel N, Limosin F, Leleu H (2014). Poor longitudinal continuity of care is associated with an increased mortality rate among patients with mental disorders: results from the French National Health insurance reimbursement database. Eur Psychiatry J Assoc Eur Psychiatr.

[35] Moscelli G, Jacobs R, Gutacker N, Aragón MJ, Chalkley M, Mason A (2019). Prospective payment systems and discretionary coding—evidence from English mental health providers. Health Econ.

[36] Häkkinen U, Iversen T, Peltola M, Seppälä TT, Malmivaara A, Belicza É (2013). Health care performance comparison using a disease-based approach: the EuroHOPE project. Health Syst Perform Comp New Dir Res Policy.

[37] Desmedt M, Vertriest S, Hellings J, Bergs J, Dessers E, Vankrunkelsven P (2016). Economic impact of Integrated Care Models for patients with chronic Diseases: a systematic review. Value Health.

[38] Del Vecchio M, Fenech L, Preti LM, Rappini V (2020). I consumi privati in sanità. Rapporto OASI 2020. Osservatorio sulle Aziende e sul Sistema Sanitario Italiano.

[39] Gottlieb L, Tobey R, Cantor J, Hessler D, Adler NE (2016). Integrating Social and Medical Data to improve Population Health: Opportunities and Barriers. Health Aff (Millwood).

[40] Fernandes S, Fond G, Zendjidjian XY, Baumstarck K, Lançon C, Berna F (2020). Measuring the patient experience of Mental Health Care: a systematic and critical review of patient-reported experience measures. Patient Prefer Adherence.

