# Supplementary material for: Using big data and Population Health Management to assess care and costs for patients with severe mental disorders and move toward a value-based payment system
Source: BMC Health Serv Res. 2023 Sep 7;23:960. doi: 10.1186/s12913-023-09655-6 (PMC10483754; doi:10.1186/s12913-023-09655-6)
Supplement: Supplementary file 1 — Supplementary Table S1: 2015-2016 SICILIA AND LAZIO ? GLM regressions for all data and selected clusters for costs, SISM costs, AHD costs. Models (M1) Full model on costs, (M2) Full model on SISM costs, (M3) SISM costs no SISM flows (M4) Costs physical health datasets (AHD) (M5) Costs AHD no AHD flows (M6) Costs Cluster 3 (M7) Costs Cluster 4 (M8) Costs Cluster 11 (M9) Costs Cluster 12 (M10) Costs Cluster 13 (M11) Costs Cluster 18 (M12) Costs Cluster 19. Supplementary Figure S1 _ Mean crude and adjusted (for models 1, 2 and 5) costs (reimbursement tariffs) and 95% confidence intervals, pooled Sicily and Lazio, years 2015-2016 - All and Clusters 3, 11, 12, 18, 19. [file 12913_2023_9655_MOESM1_ESM.docx]

Supplementary Table S1

2015-2016 SICILIA AND LAZIO – GLM regressions for all data and selected clusters for costs, SISM costs, AHD costs. Models (M1) Full model on costs, (M2) Full model on SISM costs, (M3) SISM costs no SISM flows (M4) Costs physical health datasets (AHD) (M5) Costs AHD no AHD flows (M6) Costs Cluster 3 (M7) Costs Cluster 4 (M8) Costs Cluster 11 (M9) Costs Cluster 12 (M10) Costs Cluster 13 (M11) Costs Cluster 18 (M12) Costs Cluster 19

|  | M1 | | M2 | | M3 | | M4 | | M5 | | M6 | | M7 | | M8 | | M9 | | M10 | | M11 | | M12 | |
| --- | --- | --- | --- | --- | --- | --- | --- | --- | --- | --- | --- | --- | --- | --- | --- | --- | --- | --- | --- | --- | --- | --- | --- | --- |
| Independent variables | cost | cost SISM | | cost SISM | | cost AHD | | cost AHD | | cost | | cost | | cost | | cost | | cost | | cost | | cost | |  |
| Male | 1.000 | | 1.027 | | 1.126 | | 0.971 | | 0.946 | | 1.032 | | 1.100 | | 0.889 | | 1.108 | | 0.905 | | 1.150 | | 0.848 | |
| Lazio | 1.066^*^ | | 0.748^***^ | | 1.806^***^ | | 1.818^***^ | | 1.244^***^ | | 0.977 | | 1.208 | | 0.817^*^ | | 1.181^*^ | | 1.108 | | 1.103 | | 1.285^*^ | |
| Year 2015 | 1 | | 1 | | 1 | | 1 | | 1 | | 1 | | 1 | | 1 | | 1 | | 1 | | 1 | | 1 | |
| Year 2016 | 0.955 | | 1.040 | | 1.096 | | 0.851^***^ | | 0.876^**^ | | 0.991 | | 0.995 | | 0.927 | | 0.988 | | 0.946 | | 0.957 | | 1.022 | |
| 18-24 years | 1 | | 1 | | 1 | | 1 | | 1 | | 1 | | 1 | | 1 | | 1 | | 1 | | 1 | | 1 | |
| 25-34 years | 1.068 | | 0.850^*^ | | 1.362^**^ | | 1.809^***^ | | 2.006^***^ | | 1.002 | | 1.134 | | 1.181 | | 1.048 | | 1.146 | | 0.899 | | 0.84 | |
| 35-44 years | 1.136^**^ | | 1.005 | | 1.487^***^ | | 1.642^***^ | | 2.812^***^ | | 1.253^*^ | | 1.32 | | 1.106 | | 1.203 | | 1.208 | | 1.06 | | 1.018 | |
| 65-74 years | 1.400^***^ | | 1.035 | | 1.900^***^ | | 2.228^***^ | | 4.293^***^ | | 1.453^***^ | | 2.639^***^ | | 1.247 | | 1.323^*^ | | 1.611^**^ | | 1.415^*^ | | 1.069 | |
| over 74 years | 1.644^***^ | | 1.103 | | 1.754^***^ | | 2.188^***^ | | 6.724^***^ | | 1.816^***^ | | 2.289^***^ | | 1.208 | | 2.180^***^ | | 1.475^*^ | | 1.201 | | 1.189 | |
| MCS class 1 | 1.135 | | 1.699^***^ | | 1.303 | | 1.062 | | 1.042 | | 1.603^*^ | | 0.478 | | 0.776 | | 1.156 | | 1.543^*^ | | 1.141 | | 1.134 | |
| MCS class 2 | 1.071 | | 1.428^***^ | | 1.795^***^ | | 0.903 | | 0.89 | | 1.213 | | 0.645 | | 0.993 | | 1.164 | | 1.309 | | 1.327^*^ | | 1.21 | |
| MCS class 3 | 1.044 | | 1.223^**^ | | 1.330^***^ | | 1.093 | | 1.04 | | 1.131 | | 0.651^*^ | | 1.161 | | 0.99 | | 1.262^*^ | | 1.078 | | 0.942 | |
| MCS class 4 | 1.041 | | 0.877 | | 0.617^***^ | | 0.957 | | 0.979 | | 1.113 | | 1.463 | | 1.068 | | 1.114 | | 0.941 | | 0.982 | | 0.922 | |
| MCS class 5 | 1.037 | | 0.714 | | 0.378^***^ | | 1.36 | | 1.165 | | 1.247 | | 1.111 | | 1.345 | | 0.459^*^ | | 1.481 | | 0.866 | | 2.374^**^ | |
| Time in MH database (SISM) | 1.008^***^ | | 1.010^**^ | | 1.006 | | 1.006 | | 1.006^*^ | | 1.008 | | 0.998 | | 0.998 | | 1.008^*^ | | 1.014^*^ | | 0.998 | | 0.999 | |
| Probability of correct MHCT | 0.987 | | 1.03 | | 0.976 | | 0.975 | | 0.978 | | 1.029 | | 0.773 | | 0.939 | | 0.972 | | 1.154 | | 1.132 | | 0.127^***^ | |
| Hospitalization | 2.516^***^ | | 1.07 | | 1.480^***^ | | 5.404^***^ | |  | | 2.831^***^ | | 2.590^***^ | | 2.587^***^ | | 2.494^***^ | | 2.034^***^ | | 2.520^***^ | | 2.551^***^ | |
| Emergency room visit | 0.973 | | 0.945 | | 0.879 | | 1.038 | |  | | 0.972 | | 1 | | 1.016 | | 0.982 | | 0.963 | | 1.011 | | 0.977 | |
| Ambulatory services | 1.404^***^ | | 1.169^*^ | | 1.449^***^ | | 2.533^***^ | |  | | 1.468^***^ | | 1.596^**^ | | 1.507^***^ | | 1.389^***^ | | 1.617^***^ | | 1.208^*^ | | 1.447^**^ | |
| Medication purchases | 1.639^***^ | | 0.793^**^ | | 0.750^**^ | | 13.116^***^ | |  | | 1.484^***^ | | 2.264^***^ | | 2.058^***^ | | 1.608^***^ | | 1.508^**^ | | 1.834^***^ | | 1.498^**^ | |
| Territorial MH services | 4.510^***^ | | 52.415^***^ | |  | | 1.1 | | 1.145 | | 7.386^***^ | | 3.246^***^ | | 3.574^***^ | | 4.758^***^ | | 3.322^***^ | | 5.782^***^ | | 4.094^***^ | |
| Semi-residential MH care | 8.144^***^ | | 202.718^***^ | |  | | 0.903 | | 1.357^**^ | | 8.550^***^ | | 4.127^***^ | | 6.271^***^ | | 7.191^***^ | | 6.138^***^ | | 8.345^***^ | | 11.288^***^ | |
| Residential MH care | 2.019^***^ | | 102.966^***^ | |  | | 1.133 | | 1.232^*^ | | 2.221^***^ | | 3.315^***^ | | 2.912^***^ | | 2.080^***^ | | 1.755^***^ | | 2.379^***^ | | 1.127 | |
| Schizophrenia | 1.093 | | 1.535^***^ | | 1.353^**^ | | 0.951 | | 0.85 | | 1.202 | | 0.776 | | 1.318^*^ | | 0.894 | | 0.865 | | 1.002 | | 1.046 | |
| Major depression | 1.017 | | 0.956 | | 0.504^***^ | | 1.146 | | 1.165 | | 1.260^*^ | | 0.743 | | 1.461^*^ | | 0.759 | | 0.732 | | 0.590^**^ | | 0.518^*^ | |
| Bipolar disorder | 1.126 | | 1.299^*^ | | 0.668^**^ | | 1.22 | | 1.064 | | 1.391^**^ | | 0.605^*^ | | 1.376^*^ | | 0.984 | | 0.848 | | 0.991 | | 0.579^*^ | |
| Personality disorder | 1 | | 1 | | 1 | | 1 | | 1 | | 1 | | 1 | | 1 | | 1 | | 1 | | 1 | | 1 | |
| Constant | 434.799^***^ | | 6.436^***^ | | 886.589^***^ | | 22.747^***^ | | 741.582^***^ | | 317.417^***^ | | 256.693^***^ | | 274.471^***^ | | 458.189^***^ | | 554.874^***^ | | 407.606^***^ | | 1082.710^***^ | |
| *N* | 6180 | | 6180 | | 6180 | | 6180 | | 6180 | | 964 | | 369 | | 788 | | 818 | | 358 | | 516 | | 385 | |

Exponentiated coefficients. MCS – Multisource comorbidity score; SISM- Sistema informativo salute mentale (National Mental Health Information System); MHCT – Mental Health Clustering Tool

^*^ *p* < 0.05, ^**^ *p* < 0.01, ^***^ *p* < 0.001

Supplementary Figure S1 _ Mean crude and adjusted (for models 1, 2 and 5) costs (reimbursement tariffs) and 95% confidence intervals, pooled Sicily and Lazio, years 2015-2016 - All and Clusters 3, 11, 12, 18, 19


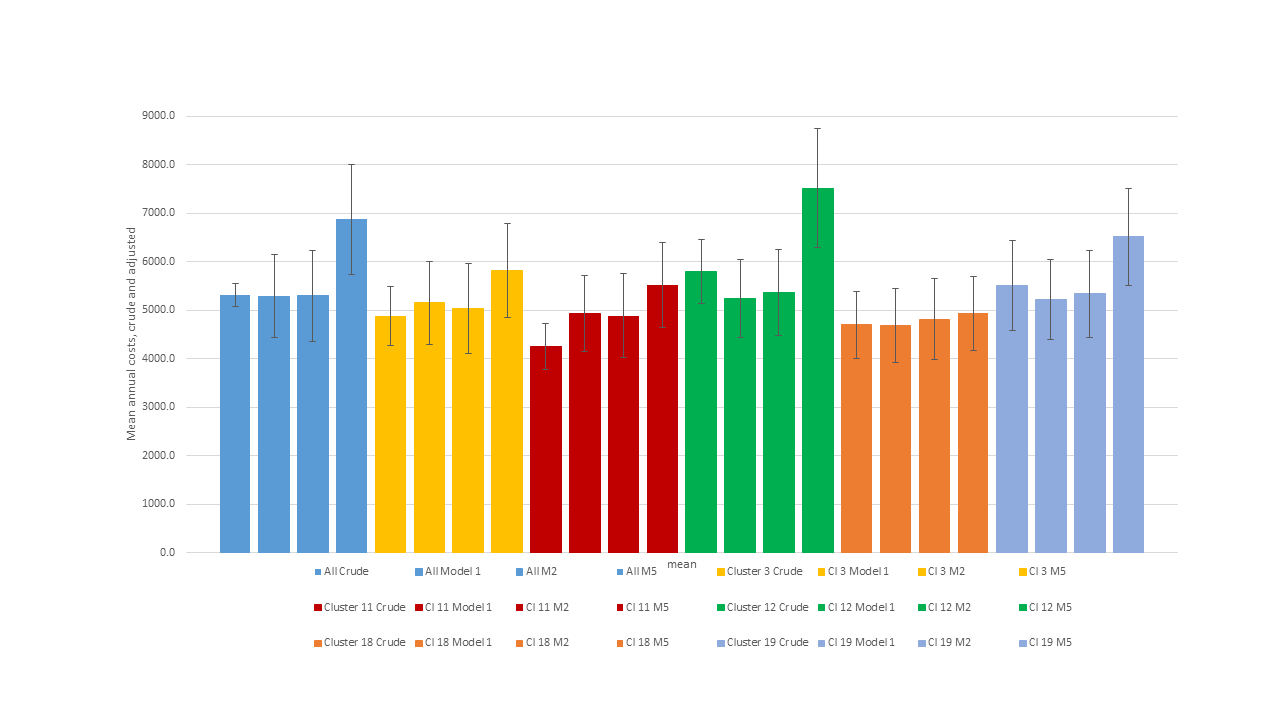


*Models: 1- Basic – controlling for gender, region, year, age class, comorbidities, 2-Basic plus diagnoses, 5-Basic plus presence in various data flows and diagnoses.
